# The Role of Temporal Order in Egocentric and Allocentric Spatial Representations

**DOI:** 10.3390/jcm12031132

**Published:** 2023-02-01

**Authors:** Tina Iachini, Francesco Ruotolo, Mariachiara Rapuano, Filomena Leonela Sbordone, Gennaro Ruggiero

**Affiliations:** Department of Psychology, Università degli Studi della Campania “L. Vanvitelli”, 81100 Caserta, Italy

**Keywords:** egocentric/allocentric spatial reference frames, temporal order, embodied spatial cognition

## Abstract

Several studies have shown that spatial information is encoded using two types of reference systems: egocentric (body-based) and/or allocentric (environment-based). However, most studies have been conducted in static situations, neglecting the fact that when we explore the environment, the objects closest to us are also those we encounter first, while those we encounter later are usually those closest to other environmental objects/elements. In this study, participants were shown with two stimuli on a computer screen, each depicting a different geometric object, placed at different distances from them and an external reference (i.e., a bar). The crucial manipulation was that the stimuli were shown sequentially. After participants had memorized the position of both stimuli, they had to indicate which object appeared closest to them (egocentric judgment) or which object appeared closest to the bar (allocentric judgment). The results showed that egocentric judgements were facilitated when the object closest to them was presented first, whereas allocentric judgements were facilitated when the object closest to the bar was presented second. These results show that temporal order has a different effect on egocentric and allocentric frames of reference, presumably rooted in the embodied way in which individuals dynamically explore the environment.

## 1. Introduction

To perform most everyday tasks, ranging from buttoning a shirt to grasping a cup to navigating the environment, we rely on two fundamental cognitive abilities, namely visual–spatial perception and spatial memory. Visual–spatial perception refers to the ability to identify and understand the position of objects in space, while spatial memory refers to the ability to store and retrieve this information in order to plan and/or perform an action [1]. There is broad consensus that we encode and represent spatial locations in memory according to two main frames of reference (FoRs): egocentric and allocentric. The egocentric FoR allows us to encode spatial information in relation to our body and is dependent on the observer’s point of view (e.g., the cup is/was one metre away from me); the allocentric FoR allows us to encode the spatial relationships between elements in the environment regardless of the observer’s position (e.g., the cup is/was one metre away from the book) [2,3,4,5].

The distinction between egocentric and allocentric FoRs is supported by behavioural and neurofunctional/neuropsychological evidence. Regarding behavioural evidence, both past and more recent studies have shown that individuals use the egocentric reference system more easily than the allocentric one. This is because the body represents the primary interface between the individual and the environment, whereas the construction of an allocentric representation requires detaching from one’s own perspective and focusing on the external environment [6,7,8]. More importantly, egocentric and allocentric representations can be selectively influenced by several environmental (e.g., environmental configuration and familiarity with the environment) [9,10,11,12], individual (e.g., age and vision abilities) [13] and task-related factors (e.g., delay between stimulus presentation and response) [14]. In addition, several studies have shown that the use of egocentric and/or allocentric representations can be selectively assessed and trained [6,15,16,17,18,19,20]. Regarding neural evidence, research has shown that the two FoRs are supported by two partially distinct brain networks, with frontoparietal areas supporting mainly the egocentric FoR (more right-sided) and occipitotemporal areas supporting mainly the allocentric FoR (hippocampal formation and lingual gyrus) [11,21,22,23,24,25,26,27,28,29,30,31,32]. Behavioural and neurofunctional findings support the idea that egocentric and allocentric representations have different functions. Specifically, egocentric spatial representations are mainly, though not exclusively, used for action with objects (e.g., to grasp an object one must primarily encode its position relative to the body), whereas allocentric representations are mainly, though not exclusively, used for recognition (e.g., in recognising a mug one needs to know where the handle is relative to the jar and not relative to the body) [33]. Recently, Ruggiero and colleagues [34] have shown that egocentric representations are particularly related to the spatial encoding of objects in action space (i.e., within 60 cm from our body), or peripersonal space, whereas allocentric representations may be more related to the extrapersonal far space (i.e., from at least one metre away from the body).

It is worth clarifying that the use of egocentric and allocentric representations is very often intertwined: if we move around in the environment, we need an update of the egocentric representation of places [35] and, due to the increase in environmental cues and landmarks, we need allocentric representations that help us to make our actions and navigation more efficient [36,37,38]. However, when we explore the environment, we are not only representing where the elements in the environment appear, but also the temporal order in which they appear. For example, when we need to reach our supermarket or our favourite restaurant, it is necessary to remember not only whether a certain building is near to us or to another one, but also which appears before or after another building and before or after us along the route. This example suggests that people can represent elements of the environment not only by considering their position in relations to spatial FoRs, but also by the temporal order in which they appear [39].

In this study, we explore the effect of temporal order on the capacity to represent spatial positions according to egocentric and allocentric FoRs. Neurofunctional evidence suggests a close relationship between spatial and temporal information processing [40,41,42,43,44]. Indeed, mediotemporal and frontoparietal areas play a key role in time perception and in encoding egocentric and allocentric FoRs [45,46,47,48,49,50,51]. Recent studies have also found that the temporal dimension is mentally represented through spatial coordinates [52,53,54,55,56,57,58,59,60,61,62,63]. Finally, it has been suggested that egocentric and allocentric spatial mechanisms underlie both the formation of implicit memories such as procedural memory (e.g., learning sequences of movements) [64] and explicit or declarative memories, such as episodic memory, that is, a type of memory in which both spatial and temporal components play a crucial role [65]. In line with this, recent studies have shown a positive relationship between egocentric navigational abilities (e.g., pathway integration) and performance in episodic memory tasks [19,20]. However, the use of egocentric and allocentric reference systems to recall the location of environmental stimuli appearing at different times has been scarcely explored [66]. So, the question arises: is egocentric/allocentric spatial encoding affected by the temporal order of appearance of memorized stimuli?

Previous studies on spatial memory used the Ego/Allo Task that requires the retrieval of a location according an egocentric and/or allocentric spatial frame [14,67,68,69]. These studies adopted a static experimental paradigm in which stimuli were presented simultaneously. Since our aim was to explore the relationship between spatial location (where) and temporal order (when), we added a temporal component to the Ego/Allo task by presenting stimuli sequentially. Therefore, we manipulated temporal order by presenting two stimuli sequentially on a virtual desk and we required spatial judgments with respect to the body and to an allocentric target (a black bar). More specifically, after both objects had disappeared, participants had to judge which stimulus was nearest to them in the “egocentric condition”, and which stimulus was nearest to the black bar in the “allocentric condition”. The stimulus nearest to the body or the bar could appear first or second.

In line with previous studies [31,67,68], we expected a general advantage of egocentric judgments over allocentric ones. Moreover, if the encoding of dynamic spatial information is influenced by what typically occurs during our exploratory activity, then we expect an advantage for egocentric encoding when the object closest to the body is presented first rather than second. Finally, we explore whether temporal information exerts a similar or different effect on the allocentric encoding.

## 2. Materials and Methods

### 2.1. Participants

Forty participants (20 females, mean age = 21.95, SD = 2.96, range = 19–29; 20 males: mean age = 24.10, SD = 2.75, range = 20–31) took part in the experiment. A sensitivity analysis was carried out with G*Power (G*power 3.1.9.2, [70,71]) and showed that with 40 participants, our test had a Power of 0.95 to reliably detect an effect size of at least 0.29 (Cohen’s f). All participants were right-handed and had normal or corrected-to-normal vision. During the initial debriefing, participants who reported neuropsychological disorders or were being treated with medication for psychological disorders were excluded from participation. All participants were recruited by word of mouth among the students of the Department of Psychology of the University of Campania. Participants were naïve to the purpose of the experiment and provided their informed consent. Recruitment and testing were in conformity with the local Ethics Committee requirements and the 2013 Helsinki Declaration [72].

### 2.2. Materials

#### 2.2.1. Spatiotemporal Ego/Allo Task

This task is a dynamic version of the static “Ego/llo Task” [67]. It takes into account two variables, space and time, by manipulating the temporal order in which egocentric and allocentric targets appeared. Therefore, participants were asked to provide egocentric and allocentric spatial judgments (“Which object was closest to you/the bar?”, respectively) and if the required target appeared first or second.

#### 2.2.2. Setting and Stimuli

The experiment was carried out in a soundproofed, comfortable room. Virtual reality task was built and administered using 3-D Vizard Virtual Reality Software Toolkit 5 (Worldviz, LLC, Santa Barbara, CA, USA). Participants were seated in a chair approximately 50 cm from the computer monitor.

The stimuli were built with the 3D modelling software Sketchup Pro 2018 based on those used by Iachini and colleagues in several previous studies [34,67,68,73,74,75,76]. They comprised four easily nameable actual geometrical objects (cube, pyramid, sphere and cone). They varied in colour (dark/medium/light grey) and size: big objects (8 × 8 cm) and small objects (6 × 6 cm). These objects were presented on a virtual table (50 × 35 × 2 cm) along with a black bar (10 × 6.6 cm).

These objects were presented in dyads along with a black bar presented on a side of the virtual table.

**Figure 1:**
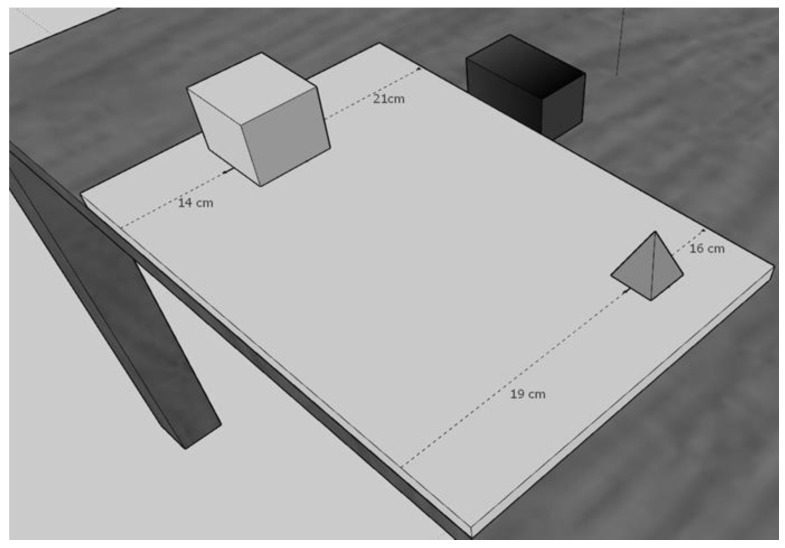
The figure shows how the 3D geometrical objects (e.g., cube and pyramid) were positioned on the panel in a way that the metric difficulty for allocentric and egocentric judgments was the same for each dyad. For example, the egocentric metric difficulty due to the cube and pyramid distances from the body was 5 cm (i.e., 19 cm–14 cm) and the allocentric metric difficulty due to the cube and pyramid distances from the black bar was also 5 cm (i.e., 21 cm–16 cm).

The objects were placed on the table at different distances with respect to the observer and the black bar. The difference between the two distances provided the metric difficulty of the dyad for egocentric and allocentric judgments. For example, if object X was at 8 cm and object Y was at 13 cm from the participant, this meant that the metric difficulty for the egocentric judgment was 5 cm (13 − 8 = 5). For the same dyad the allocentric judgment had the same difficulty, this meant that object X was at 22 cm and object Y at 17 cm from the black bar (22 − 17 = 5 cm). Three levels of metric difficulty were used: easy = 11 cm; medium = 8 cm; difficult = 5 cm (see Figure 1).

Based on these constraints, 80 dyads were created: 40 dyads with cube and pyramid; 40 dyads with sphere and cone. The 80 dyads were presented in four blocks. Each block corresponded to a specific dyad, that is cube–pyramid or cone–sphere, and a specific spatial judgment, egocentric or allocentric. Each block included 20 dyads: 16 spatial judgments and 4 distractor questions.

### 2.3. Procedure

Participants were provided with verbal and written instructions about the experimental procedure and task. They were instructed to memorize the dyads that would be shown one at a time and to answer two different questions: “What stimulus was closest to you?” (egocentric question)—“What stimulus was closest to the bar?” (allocentric question). The kind of question was indicated by a word on the screen. The words were the following: “YOU” was for “What stimulus was near to the body”—“BAR” was for “What stimulus was near to a black bar”.

Training phase: After the instructions, participants were presented with each object and asked to name them. In this way, difficulties and errors due to naming problems could be excluded a priori. Then, participants were trained to use specific keys to provide their response: S for sphere, C for cube; C for cone and P for pyramid. The keys were highlighted on the central part of the keyboard according to a vertical dimension to avoid laterality effects and with the remaining part of the keyboard hidden. Afterwards, participants could start a training session during which they had to provide spatial judgments for 6 dyads (the presented dyads were not included in the testing phase).

Testing phase and Experimental Design: The experiment was organized in blocks. Each block corresponded to a specific spatial judgment and a specific dyad. There were four blocks: ego cube–pyramid; ego cone–sphere; allo cube–pyramid; allo cone–sphere. Blocks were presented in counterbalanced order. Within each block, the order of presentation of trials was randomized. Within the egocentric block, participants had to provide an egocentric spatial judgment (“What object was closest to you”). Within the allocentric block, participants had to provide an allocentric spatial judgment (“What object was closest to the bar”). Each block comprised 16 trials regarding egocentric/allocentric spatial judgments and four distractor questions (total: 64 trials + 16 distractors). The testing phase was administered once for each participant.

We planned a random presentation of distractors in each block, asking participants which stimulus was the tallest one. Distractors prevented participants from understanding the ultimate purpose of the experiment.

**Figure 2:**
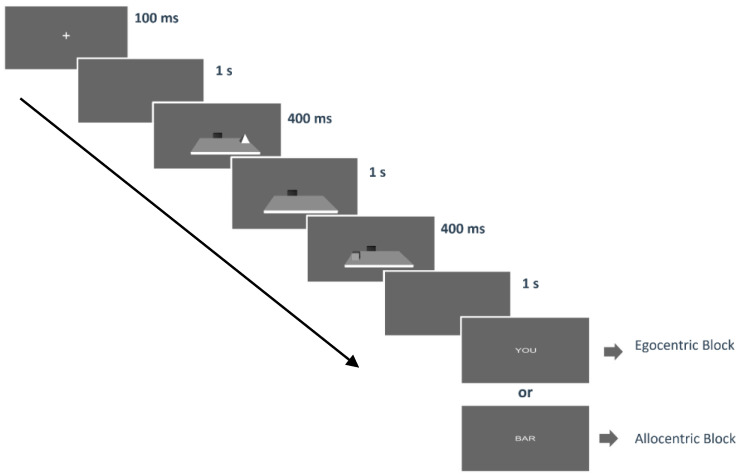
The figure shows a schematic overview of one trial. Each trial started with a fixation cross (100 ms) followed by a blank screen. After 1 s, the first object appeared for 400 ms. It could be the nearest to the body or the bar. After that, only the panel with the black bar remained. Afterwards, the second stimulus would appear for another 400 ms: this could be the nearest to the body or the black bar. Finally, the virtual desk disappeared and after a 1 s blank, the word indicating the corresponding question (“you”, “bar”) appeared. The arrow indicates the experimental flow.

Each trial started with the presentation of a fixation cross on a grey screen for 100 ms; immediately after, a blank screen was presented for 1 s; then, the first object appeared for 400 ms and it could be nearest to the body (egocentric condition first) or to the bar (allocentric condition first). Afterwards, the second object appeared for 400 ms, and it could be nearest to the body (egocentric condition second) or to the black bar (allocentric condition second). Then, the virtual desk disappeared and after a 1 s blank, the word indicating the spatial judgement (“you” for egocentric, “bar” for allocentric) appeared (see Figure 2). Participants were instructed to respond as accurately and quickly as possible, although there were no time limits. Mean accuracy and RTs measured the performance.

## 3. Results

### 3.1. Data Analysis

Preliminary descriptive analyses were performed to assess normality and outliers [77]. Data > ±2.5 SDs were discarded (corresponding to 2.5% of the data). Mean accuracy (accurate = 1, wrong = 0) and mean RTs of correct responses for each condition (ego first, ego second, allo first, allo second) were calculated. The skewness and kurtosis values of the accuracy data indicated that the distribution was normal (average skewness = −0.46, range: −0.25 to −0.54; average kurtosis = −0.88, range: −0.71 to −1.16) [78]. Similarly, the skewness and kurtosis values of the RTs data indicated that the distribution was normal (average skewness = 0.56, range: 0.49 to 0.678; average kurtosis = 0.22, range: −0.43 to 0.33). In order to assess whether temporal order influenced the use of egocentric and allocentric reference systems, a within-subject ANOVA with spatial frames as a 2-level factor (Ego/Allo) and temporal order as a 2-level factor (first–second) was used on participants’ accuracy and response time, respectively. The Bonferroni test was used to analyse post-hoc effects and the magnitude of effect size was expressed by *η*^2^*_p_*. Finally, a Pearson correlation analysis between accuracy and response time was performed to check for the possible presence of a confounding factor in the data due to the speed–accuracy trade-off.

### 3.2. Results from the ANOVA

#### 3.2.1. Accuracy

**Figure 3:**
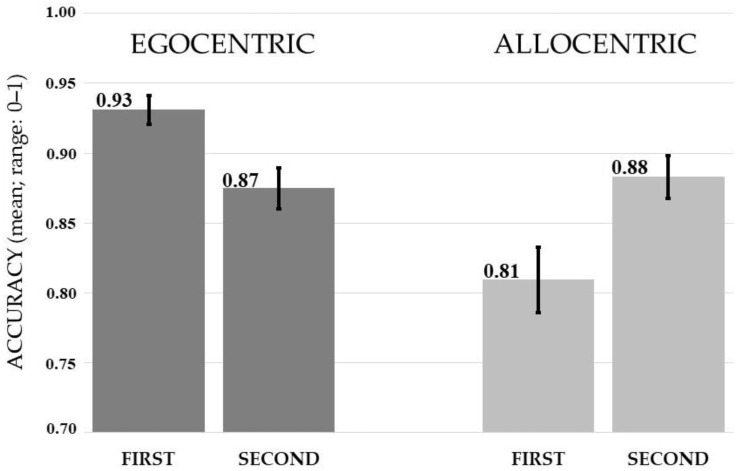
The graph shows the mean accuracy of egocentric and allocentric judgments as a function of the order of presentation of the stimuli (i.e., first vs. second). Vertical bars represent standard errors.

The ANOVA showed a significant main effect of spatial frame (*F*(1, 39) = 9.334, *p* = <0.005, *η*^2^*_p_* = 0.19). Egocentric judgments (*M* = 0.90, *SD* = 0.79) were more accurate than allocentric ones (*M* = 0.85, *SD* = 0.12). A significant spatial frame x temporal order interaction emerged: *F*(1, 39) = 22.382, *p* < 0.0001, *η*^2^*_p_* = 0.36. The Bonferroni post-hoc test showed that the interaction was due to the opposite effect of temporal order on frames of reference: while the egocentric encoding was facilitated if the target closest to the body appeared first (*M* = 0.93, *SD* = 0.06), the allocentric encoding was facilitated if the target closest to the external mark (i.e., black bar) appeared second (*M* = 0.88, *SD* = 0.10). In fact, egocentric judgements were more accurate when the target appeared first than second (*M* = 0.87, *SD* = 0.09) (*p* < 0.05), while the allocentric first judgements (*M* = 0.81, *SD* = 0.15) were less accurate than all other combinations (at least *p* < 0.01) (see Figure 3).

#### 3.2.2. Response Time (RT)

**Figure 4:**
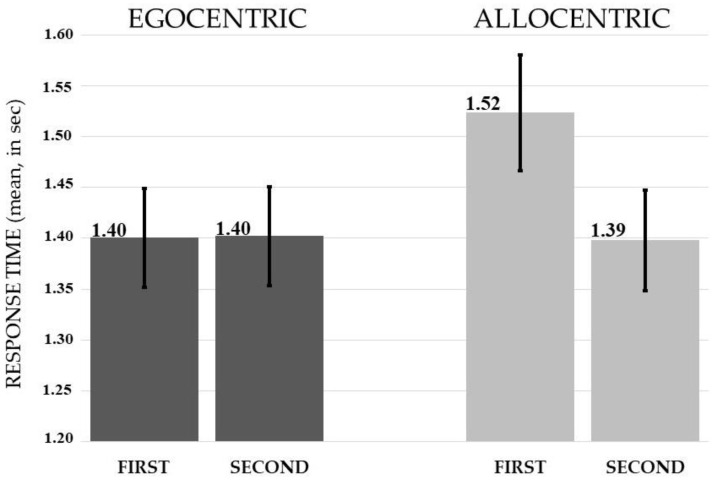
The graph shows the mean response time of egocentric and allocentric judgments as a function of the order of presentation of the stimuli (i.e., first vs. second). Vertical bars represent standard errors.

The ANOVA showed a significant main effect of the temporal order (*F*(1, 39) = 4.17, *p* = <0.05, *η*^2^*_p_* = 0.10). Judgments provided on the objects that appeared first (*M* = 1.46, *SD* = 0.33) were slower than judgments provided on the objects that appeared second (*M* = 1.40, *SD* = 0.31). A significant spatial frame x temporal order interaction also emerged: *F*(1, 39) = 4.29, *p* < 0.05, *η*^2^*_p_* = 0.10. The Bonferroni test showed an effect of temporal order only on allocentric judgments: the performance was slower than all other conditions if the target closest to the black bar appeared first (allo first *M* = 1.52, *SD* = 0.36; allo second *M* = 1.40, *SD* = 0.31) (at least *p* < 0.05). Instead, there was no significant difference between egocentric judgments (ego first *M* = 1.40, *SD* = 0.31; ego second *M* = 1.40, *SD* = 0.31) (*p* = 1) (see Figure 4).

### 3.3. Results from the Correlation Analysis

No statistically significant correlations appeared (i.e., all p_s_ at least > 0.05) between the mean of accuracy and RTs (*r* range values: *r* = 0.05 to *r* = 0.24). Therefore, no speed–accuracy trade-off effect emerged.

## 4. Discussion

This study reports preliminary data about the influence of temporal order on the retrieval of spatial locations according to egocentric and allocentric frames of reference. Participants saw two objects appearing one after the other on a panel and memorized their position. Once the objects disappeared, participants judged which object was closer to them (egocentric judgment) or to a black bar (allocentric judgment). Overall, the results from the ANOVA showed that participants were more accurate with egocentric than allocentric judgements. This is in line with previous evidence showing an advantage for body-centred egocentric representations over object-centred allocentric representations ([25,67] for reviews, see [79,80]). This may reflect the primacy of egocentric organization of spatial information that can be attributed to our natural egocentrically mediated interface with the environment (e.g., [6,7]).

More interestingly, the innovative result of the current study comes from the interaction effect between egocentric/allocentric reference frames and the temporal order of object presentation. Results showed that the order in which stimuli appeared had an opposite effect on frames of reference: while the egocentric encoding was more accurate if the target object closest to the body appeared first than second, the allocentric encoding was more accurate if the target object closest to the black bar appeared second rather than first. In terms of response time, results showed that judgments provided on the objects that appeared first were slower than judgments provided on the objects that appeared second. This result could reflect the interaction effect. Indeed, the results revealed that temporal order only affected the allocentric judgments: they were slower than all other judgments when the stimulus closer to the black bar appeared first. No significant difference between the egocentric judgments appeared.

A possible explanation of the advantage of egocentric judgments when the related target object appeared first than second can be rooted in the nature of the task used for the current study. In fact, all stimuli were displayed in the participant’s peripersonal space, that is, the space that directly surrounds us and which we can act upon. In this regard, Ruggiero and colleagues [34] showed that when the geometric stimuli of the Ego/Allo task were presented in the peripersonal space and participants had their arms bended behind their back, egocentric judgements were less accurate than when participants had their arms free. This motor interference effect did not appear when the objects were presented in the extrapersonal space (i.e., objects located in that part of space could not be grasped just by extending one’s arm). This suggests that we construct a spatial representation of objects in peripersonal space starting from the body as the centre of action possibility. More specifically, in this space of manipulation, where we can explore the environment simply by extending our arms, the object we touch first is also the closest one. This could lead to forming a kind of natural association between what we encounter first and what is closest. This explanation is reinforced by several studies that show that as soon as an object enters our peripersonal space, it immediately attracts our attention and we prepare to act with it (e.g., [81,82]).

Additionally, the possibility that the use of egocentric reference systems is linked to both spatial and temporal aspects has been recently supported by Committeri and colleagues [19,20]. Specifically, they have shown that the accuracy in egocentric navigation (i.e., path integration performance) predicts participants’ performance in episodic memory tasks that by their nature require both spatial (where an event occurred) and temporal (when an event occurred) aspects. More recently, it has also been shown that a training focused on egocentric navigation improves performance in episodic memory tasks [83]. Thus, the results of the present study further enrich the evidence on the relationship between spatial reference systems and temporal features, highlighting how the order of appearance of events modulates the use of an egocentric system.

However, how can we explain the allocentric advantage when the target object appeared second? We could argue that information retrieval happens in few seconds and thus we are within visuospatial short-term memory [84,85]. Several studies about serial recall of items have suggested a facilitation for the last-learned items in short-term memory (i.e., recency effect). Although our task involved only two stimuli, we might think that facilitation for the last target object simply reflects this short-term memory mechanism. However, an alternative explanation is possible. In our setting, if we consider the body as the centre of spatial exploration and organization, the black bar constitutes the anchorage point furthest from the body. Thus, it is possible that we naturally form an association between what we encounter last and what is furthest away. This possible interpretation is consistent with the egocentric advantage for the first target object.

In sum, the “body in action” interpretation is in line with an embodied and situated perspective of spatial cognition [86] and with the idea that spatial encoding involves a motor aspect [87]. According to the philosopher and mathematician Poincaré [88], localizing an object in space “…simply means that we represent the movements that are necessary to reach that object…” (p. 75). Therefore, the body in space defines both spatial and temporal relationships. However, this speculation needs further research in the future. Moreover, future studies could address the topic of individual differences since differences due to gender, cultural or motivational factors are often reported in the spatial cognition literature [89]. For example, a better male performance in spatial tasks requiring allocentric frames is often reported and motivational factors (such as the perceived individual ability to cope with the spatial task) seem to play a crucial role in these effects [90,91].

From a clinical point of view, the task used in this study shows interesting application possibilities. For example, it has been shown that the decline in visual–spatial abilities in patients with amnestic mild cognitive impairment may represent an early marker of the Alzheimer’s disease conversion [76]. Moreover, episodic memory problems, as well as spatial disorientation, are very common in patients with Alzheimer’s dementia [92]. In other words, the prodromal signs of conversion from MCI to Alzheimer’s dementia could be traced precisely in the inability to spatially and temporally encode events. For this reason, it is crucial to have reliable, sensitive and easy-to-administer tasks to detect early signs of cognitive impairment. In this regard, even if the task used in the current study does not reflect the complexity of navigable environments, it has the advantage of helping us to identify in a simple way the basic mechanisms of spatial abilities [76]. In fact, it is possible that the dynamic condition implemented in the Ego/Allo task captures aspects of route-type spatial representations in which we organize spatial environmental elements/landmarks according to spatiotemporal sequences in relation to bodily exploration [19,93]. Future studies should investigate the impact of healthy and pathological aging on the ability to use egocentric and allocentric frames of reference, also taking into consideration temporal aspects.

## Data Availability

The data presented in this study are available on request from the corresponding author.
